# Supplementary material for: Complex‐centric proteome profiling by SEC‐SWATH‐MS
Source: Mol Syst Biol. 2019 Jan 14;15(1):e8438. doi: 10.15252/msb.20188438 (PMC6346213; doi:10.15252/msb.20188438)

Q9Y375
Annotated subunits: 11   Subunits with signal: 9
Max. coeluting subunits: 7   Max. completeness: 0.64

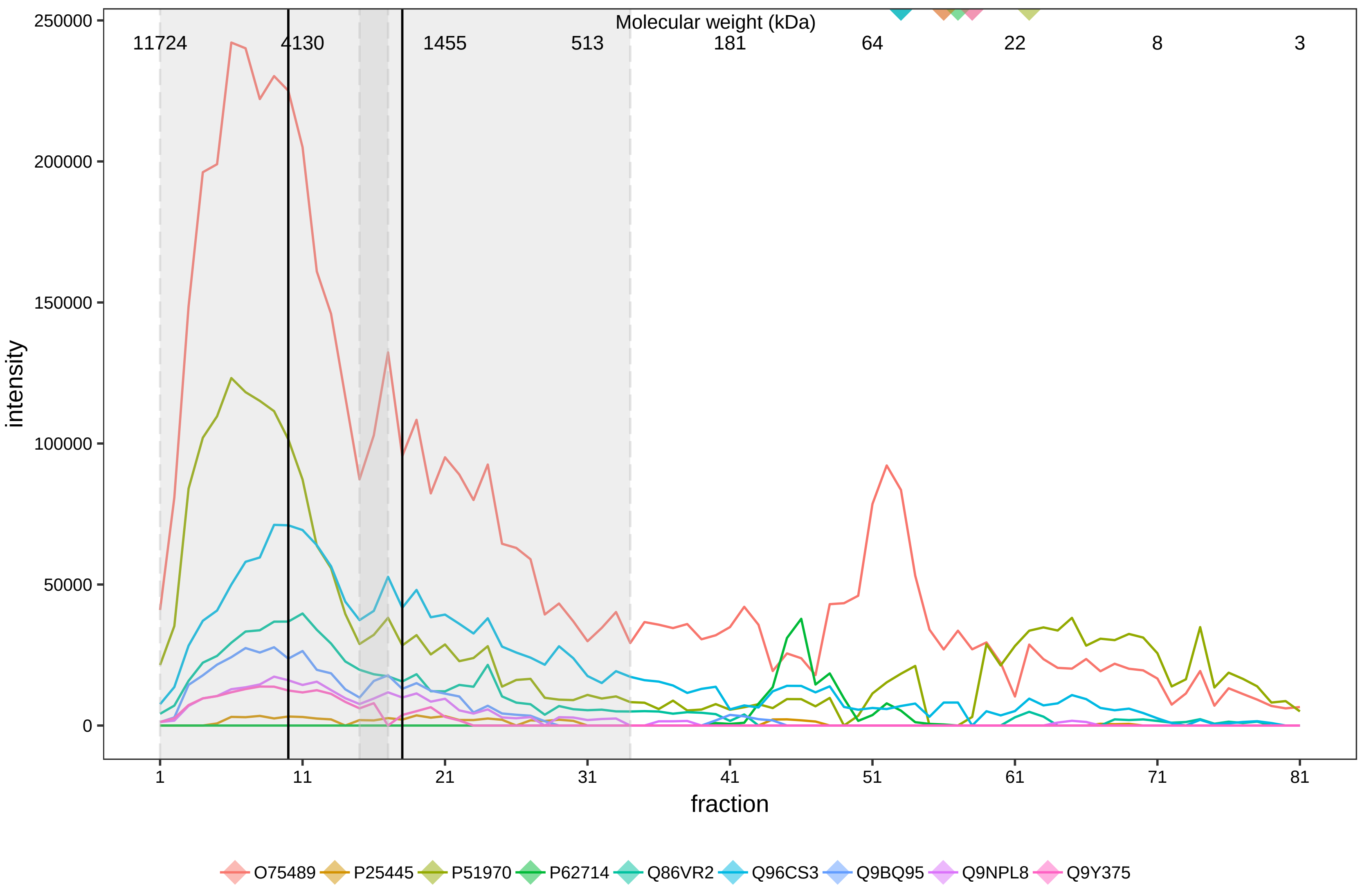

Supplement: Supplementary file 7 — Dataset EV6 [file MSB-15-e8438-s007.zip › feature_plots_bioplex/Q9Y375.pdf]
